# Stratified analysis of the correlation between gestational weight gain and birth weight for gestational age: a retrospective single-center cohort study in Japan

**DOI:** 10.1186/s12884-019-2563-5

**Published:** 2019-11-04

**Authors:** Noriko SATO, Naoyuki MIYASAKA

**Affiliations:** 1 Department of Molecular Epidemiology (Epigenetic Epidemiology), Medical Research Institute, Tokyo Medical and Dental University (TMDU), 1-5-45, Yushima, Bunkyo-ku, Tokyo, 113-8510 Japan; 2 Comprehensive Reproductive Medicine, Graduate School, Tokyo Medical and Dental University (TMDU), 113-8510, Japan, 1-5-45, Yushima, Bunkyo-ku, Tokyo, Tokyo 113-8510 Japan

**Keywords:** Birth weight for gestational age, Gestational weight gain, Body mass index, Stratum-specific correlation, Sibling comparison

## Abstract

**Background:**

Japan has an exceptionally high proportion of low-weight births and underweight women. It has been suggested that an appropriate increase in gestational weight gain (GWG) for underweight women will help to prevent low birth weight. The current strategy aims to raise the desired value of GWG equally for all pregnant women within the underweight category. However, it remains elusive whether or not the relationship between GWG and birth weight for gestational age (BW/GA) are uniformly equivalent for all the women.

**Methods:**

We performed a retrospective cohort analysis of women who delivered their newborns at Tokyo Medical and Dental University Hospital from 2013 to 2017. First, in order to examine the direct effect of an increase or decrease in GWG on BW/GA, we analyzed the correlation between inter-pregnancy differences in GWG and BW/GA using a sub-cohort of women who experienced two deliveries during the study period (*n* = 75). Second, we dichotomized the main cohort (*n* = 1114) according to BW/GA to verify our hypothesis that the correlation between GWG and BW/GA differs depending on the size of the newborn.

**Results:**

The inter-pregnancy difference in BW/GA was not correlated with that of GWG. However, the correlation between BW/GA of siblings was high (r = 0.63, *p* = 1.9 × 10^− 9^). The correlation between GWG and BW/GA in women who delivered larger-sized newborns was higher (r = 0.17, *p* = 4.1 × 10^− 5^) than that in women who delivered smaller-sized newborns (r = 0.099, *p* = 1.9 × 10^− 2^). This disparity did not change after adjustment for pre-pregnancy BMI. The mean birth weight in the dichotomized groups corresponded to percentile 52.0 and 13.4 of the international newborn size assessed by INTERGROWTH-21st standards.

**Conclusions:**

In our study, GWG was positively correlated with BW/GA for heavier neonates whose birth weights were similar to the average neonatal weight according to world standards. However, caution might be required for low-birth-weight neonates because increased GWG does not always result in increased birth weight.

## Background

Weight gain management during pregnancy is important for the future health of pregnant women and their newborns. In Japan, the high proportion of newborns with low birth weight and the high prevalence of underweight women are major problems. Low birth weight increases the risks of metabolic disorders, coronary heart disease, and other non-communicable diseases. Due to the exceptionally high proportion of low birth weight infants, there has been much focus on the establishment of a strategy to increase birth weight in Japan. It has been argued that the recommended weight gain limits during pregnancy might be too strict [1]. One promising method to prevent low-weight births may be to increase weight gain during pregnancy. A previous study established that the optimal gestational weight gain (GWG) varies largely according to the pre-pregnancy body mass index (BMI) and claimed that the current values recommended by the Japanese guidelines are lower than the optimal GWG for underweight women [2]. Conventionally, it is considered that the same optimal set point of GWG can be applied to all the pregnant women within a similar pre-pregnancy BMI category [3, 4]. However, it remains unclear whether an increase in birth weight for gestational age (BW/GA) could be equally expected as a result of a commonly designed increase in GWG.

Birth weight is a multifactorial phenotype and individuals have different multifactorial situations. Furthermore, GWG could interact with multiple other factors. Theoretically, pure GWG effects on BW/GA can be estimated by the relationship between GWG and BW/GA for the same mother. Observationally, the effects of a GWG difference on BW/GA difference between siblings could provide useful information on the direct effect of GWG [5, 6]. For example, when the same mother delivers twice and there is a difference in GWG between the two deliveries, the inter-pregnancy difference of GWG should correlate well with that of BW/GA if the increase in GWG contributed to an increase in BW/GA. However, there is a paucity of evidence regarding this in Japan.

Moreover, raising the limit of GWG with the aim to reduce low-weight births has been considered. However, if population heterogeneity exists, in which an increase in GWG may be ineffective in women who will deliver small neonates, this aim may be invalid. Therefore, the purpose of this study was to present evidence that an increase in GWG does not always contribute to an increase in BW/GA and to prompt a reconsideration of the risks and appropriateness of a uniform recommendation for GWG increase. This was done with the assumption that there are two types of fetuses: those that will increase in birth weight in response to maternal weight gain (GWG responders) and those that will not increase in birth weight, even if the mother has a substantial gain in weight (non-responders). Our hypothesis was that larger-sized newborns would be GWG responders whereas smaller-sized newborns would be non-responders.

## Methods

### Study setting and population

We performed a retrospective cohort analysis of pregnant women who delivered their newborns at Tokyo Medical and Dental University Hospital from 2013 to 2017 (*n* = 2238). For all patients, maternal and neonatal information were recorded and routinely validated in the standard electronic prenatal medical records by trained medical personnel. From these electronic prenatal medical records, all data were anonymously collected, including the following information: maternal height, maternal age, pre-pregnancy weight (self-reported), parity, gestational weight, obstetrics history (preterm birth and stillbirth), present pregnancy complications (such as hypertensive disorder of pregnancy and preterm labor), history of non-communicable diseases (such as asthma, autoimmune disorders, cancer, diabetes mellitus, hematologic disorders, renal diseases, thyroid diseases, hypertension, psychiatric disorders), child’s sex, gestational age at delivery, and neonatal body size. Gestational age was determined according to the date of the mother’s last menstrual period and confirmed via measurement of the fetal crown-rump length on a first-trimester scan. Maternal weight measurements during gestation were performed 11.3 ± 3.1 times for each woman. Institutional review board approval was obtained from Tokyo Medical and Dental University (approval number: M2017–337; May 8, 2018). The requirement for individual informed consent was waived by the institutional review board because of the retrospective nature of the study and because the data of the involved participants were anonymized. All methods were performed in accordance with relevant guidelines and regulations.

### Inclusion criteria

The eligibility criteria were as follows: Japanese singleton delivery with available pre-pregnancy BMI data and live birth, without malformation completing more than 28 weeks of gestation at the time of delivery. Exclusion involved maternal age of less than 20 years.

**Fig. 1:**
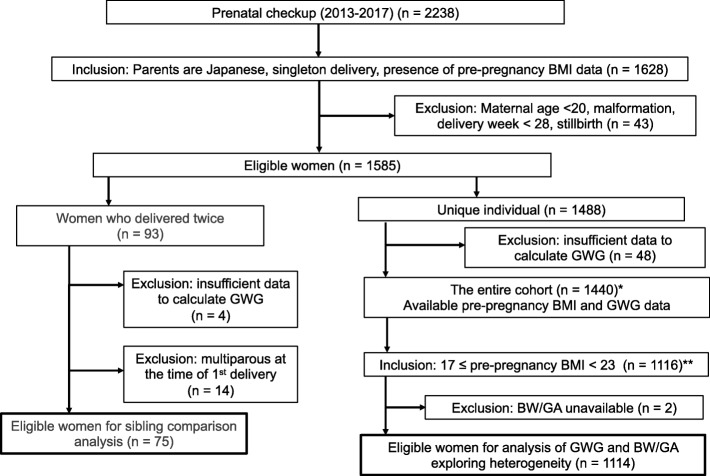
Flow diagram of the study population. *, data characteristics are shown in Additional file 3: Table S3; **, results of the data analysis are shown in Fig. 4. BMI, body mass index; BW/GA, birth weight for gestational age; GWG, gestational weight gain.

We extracted two sub-cohorts from this population for our analyses. The first involved a sibling comparison of the first and second pregnancies of each woman (75 women). This comparison was performed to assess prenatal factors that affect neonatal health to eliminate potential confounding factors such as genetics, sociodemographic factors, and other unmeasured factors. The sibling comparison was restricted to women who were primiparous at the time of first delivery during the study period. The second analysis focused on the general population to investigate the heterogeneity in terms of GWG responsiveness. Unique (non-overlapping) individuals were included in the study by selecting only one delivery for each woman with multiple deliveries during the study period. The number of women with sufficient weight data to calculate GWG was 1440; therefore, 1440 women were eligible for this study (Fig. 1).

### Gestational weight gain

GWG was calculated as the difference between maternal weight at 40 weeks of gestation and self-reported pre-pregnancy weight. Maternal weight at 40 weeks was obtained via an observed clinical measurement. In other cases when the mother did not deliver at 40 weeks, a predicted value of GWG at 40 weeks was calculated from the rate of weight gain as described previously [2]. The rate of weight gain was calculated for each woman based on the simple linear regression model for the relationship between gestational week and gestational weight. All data included in our analysis had good fitness in the simple linear regression model.

### Birth weight percentiles for gestational age, sex, and parity based on national standards

The BW/GA percentile was calculated using the Japanese neonatal anthropometric charts, which are specific to gestational age, child sex, and parity [7]. Concerning post-term deliveries (42 and 42.1 weeks of gestation observed for two neonates), the BW/GA percentile could not be calculated because the reference charts includes only gestational ages less than 42 weeks.

### Newborn size for gestational age and sex based on international standards

The international percentile value for newborn size was evaluated using INTERGROWTH-21st standards, which are specific to gestational age and sex [8].

### Data analysis

All calculations and graphical presentations were performed using R software (version 3.5.0; R Foundation for Statistical Computing, Vienna, Austria). Sibling correlation, which compared the first and second pregnancies for the same mother (*n* = 75), was estimated using the Pearson correlation coefficient (r). A minimum sample size of 32 was estimated for the correlation analysis using Sample Size Calculators (https://www2.ccrb.cuhk.edu.hk/stat/epistudies/reg1.htm), with an alpha value of 0.05, power of 0.80, and Pearson correlation coefficient of 0.48. A linear regression analysis of BW/GA percentile with respect to pre-pregnancy BMI was performed for women (*n* = 1114), with a BMI of 17 to 23 kg/m^2^. A minimum sample size of 404 was estimated for the simple linear regression analysis using Sample Size Calculators (https://www2.ccrb.cuhk.edu.hk/stat/epistudies/reg1.htm) with an alpha value of 0.05, power of 0.80, and effect size of 2.86. To analyze the heterogeneity of neonatal birth weight response to GWG, we dichotomized the women according to their BW/GA percentile: group 1 comprised women with larger-sized newborns (*n* = 557; BW/GA percentile ≥50), whereas group 2 comprised women with smaller-sized newborns (n = 557; BW/GA percentile < 50). Since the BW/GA reference was available for only gestational ages less than 42 weeks, two post-term deliveries were excluded in this analysis, which resulted in the main sample size of 1114. The difference in the correlation between GWG and BW/GA between large and small newborns was explored, both unadjusted and adjusted for pre-pregnancy BMI.

## Results

### Sibling comparison

The characteristics of our sibling cohort are shown in Table 1. Additional file 1: Table S1 and Additional file 2: Table S2 show that data were balanced for child sex and small-for-gestational age or large-for-gestational age status between the two pregnancies.

**Table 1:** Characteristics of the two deliveries (*n* = 75)

	1st delivery	2nd delivery
Maternal age (year), mean (SD)	31.1 (4.6)	33.5 (4.6)
Maternal height (cm), mean (SD)	158.6 (5.8)	158.6 (5.8)
Pre-pregnancy weight (kg), mean (SD)	51.7 (6.8)	52.1 (6.4)
Pre-pregnancy BMI (kg/m^2^), mean (SD)	20.5 (2.4)	20.7 (2.3)
Multiparity, *n* (%)	0 (0.0)	75 (100)
Gestational week of delivery, mean (SD)	39.7 (1.1)	39.1 (1.1)
Gestational weight gain (kg/40 weeks), mean (SD)	10.9 (3.3)	10.8 (2.9)
Child sex: male, *n* (%)	41 (55)	36 (48)
Birth weight (g), mean (SD)	3043 (341)	3000 (342)
Birth length (cm), mean (SD)	50 (1.8)	50 (1.8)
Head circumference (cm), mean (SD)	33 (1.3)	34 (1.1)
Chest circumference (cm), mean (SD)	32 (1.4)	32 (1.6)
BW/GA percentile, mean (SD)	53.7 (27.9)	47.9 (26)
SGA, *n* (%)	5 (6.7)	5 (6.7)

Data are presented as mean (SD) or n (%). SGA, small for gestational age.

**Fig. 2:**
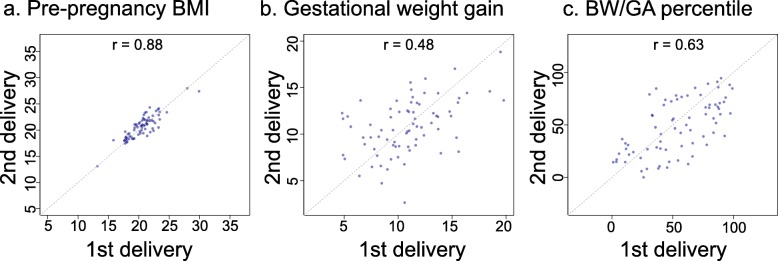
Correlations of pre-pregnancy body mass index, gestational weight gain, and birth weight for gestational age percentiles. Scatter plots showing the (**a**) pre-pregnancy BMI, (**b**) gestational weight gain, and (**c**) BW/GA percentile relationships between the two pregnancies. BMI, body mass index; BW/GA, birth weight for gestational age.

**Fig. 3:**
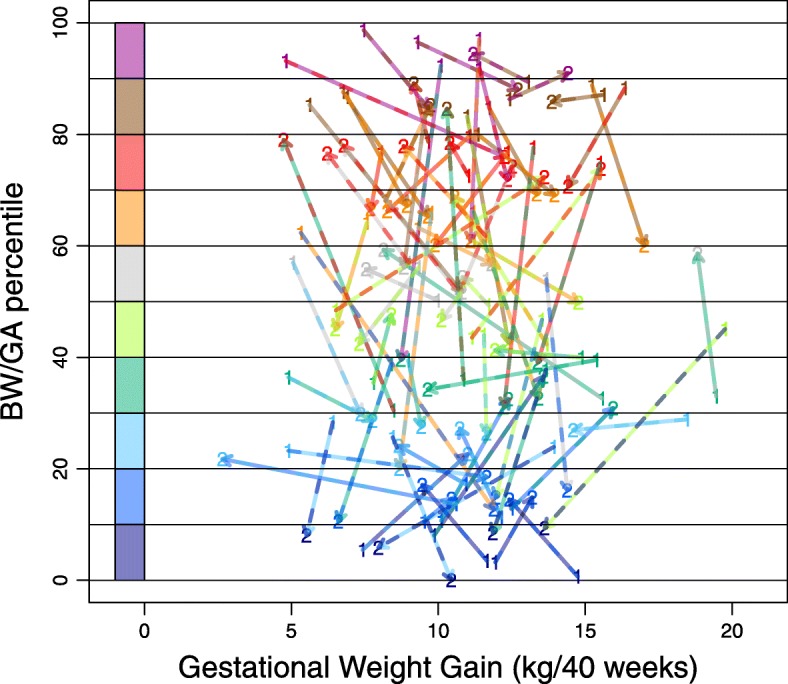
Visualization of inter-pregnancy changes in gestational weight gain and birth weight for gestational age percentiles. Scatter plot showing the relationship between gestational weight gain (GWG) and birth weight for gestational age (BW/GA) percentiles for the first and second deliveries: 1 indicates the first delivery and 2 indicates the second delivery. The “1” and “2” points are connected with arrows. Each arrow represents a unique individual. Color codes showing distinct BW/GA percentile ranges are indicated on the left. BMI, body mass index; BW/GA, birth weight for gestational age; GWG, gestational weight gain.

Differences in pre-pregnancy BMI, GWG, and BW/GA between the first and second deliveries of the same woman were examined (Fig. 2). The correlation between the first and second pre-pregnancy BMI values was high (Pearson correlation coefficient, r = 0.88; *p* < 2.2 × 10^− 16^), suggesting that the inter-pregnancy difference in BMI was negligible. In contrast, the correlation between the first and second GWG values was modest (r = 0.48; *p* = 1.2 × 10^− 5^), although the sibling correlation was high for the BW/GA percentile (r = 0.63, *p* = 1.9 × 10^− 9^), indicating that BW/GA may not be correlated with GWG. Subsequently, we investigated how GWG changes between the two pregnancies influenced differences in birth outcomes. In Fig. 3, an arrow appears between the first set and second set of GWG and BW/GA percentile data for each woman. The up- and right-ward arrow indicates an increase in both GWG and BW/GA, whereas the down- and right-ward arrow indicates a decrease in BW/GA in spite of an increase in GWG. The down- and left-ward arrow indicates a decrease in both GWG and BW/GA, whereas the up- and left-ward arrow indicates an increase in BW/GA in spite of a decrease in GWG. As a result, there was no consistent positive correlation between GWG and BW/GA as shown in Fig. 3. Overall, difference in GWG was not correlated with the difference in BW/GA (r = − 0.10; *p* = 3.8 × 10^− 1^) (Additional file 4: Figure S1a). Consistent with the high sibling correlation for the BW/GA percentile, the BW/GA percentile did not demonstrate a change of more than 30 percentiles between the two pregnancies for 80% of women (Additional file 4: Figure S1b). These results indicated that BW/GA might be largely preset before the perinatal period and GWG increase did not always contribute to the increase in BW/GA in multiple deliveries by the same mother.

### Analysis exploring heterogeneity

**Fig. 4:**
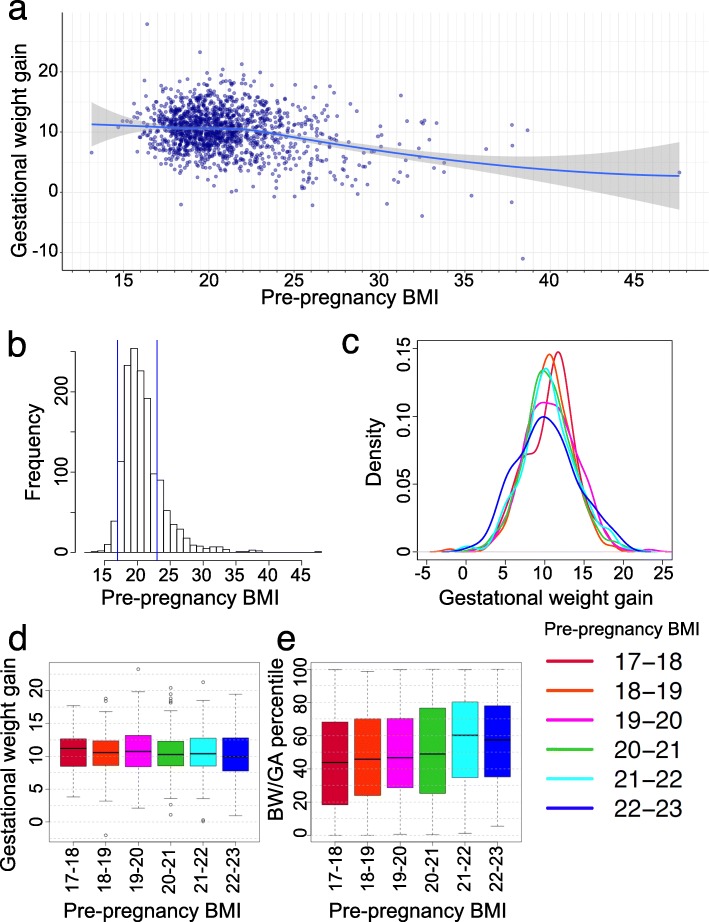
Distribution of pre-pregnancy body mass index and gestational weight gain. **a** Scatter plot shows the distribution of the mothers’ data for pre-pregnancy BMI and gestational weight gain. A smoothing line with locally estimated scatter plot smoothing is drawn in blue (confidence interval in gray). **b** Histogram of pre-pregnancy BMI. Blue lines indicate 17 and 23 kg/m^2^. **c** Density plots of gestational weight gain in each pre-pregnancy BMI category. **d** GWG differences among six groups classified according to pre-pregnancy BMI categories. **e** Differences in BW/GA percentiles among six groups classified according to pre-pregnancy BMI categories. Pre-pregnancy BMI categories: 17-18, 17 ≤ pre-pregnancy BMI < 18 (red) (n = 113); 18-19, 18 ≤ pre-pregnancy BMI < 19 (orange) (n = 233); 19 ≤ pre-pregnancy BMI < 20 (magenta) (n = 252); 20-21, 20 ≤ pre-pregnancy BMI < 21 (green) (n = 228); 21-22, 21 ≤ pre-pregnancy BMI < 22 (cyan) (n = 192); and 22-23, 22 ≤ pre-pregnancy BMI < 23 (blue) (n = 98). Box and whisker plots illustrate medians, minimums, maximums, and interquartile ranges. BMI, body mass index; BW/GA, birth weight for gestational age

We surveyed the data distribution of pre-pregnancy BMI and GWG for the unique (non-overlapping) individuals (*n* = 1440) (Fig. 4). Additional file 3: Table S3 presents the characteristics of the entire cohort according to the pre-pregnancy BMI category. Because the numbers of women in the lowest and highest BMI categories (BMI < 17 kg/m^2^, *n* = 52; BMI ≥ 23 kg/m^2^, *n* = 272) were too small for a statistically meaningful analysis of the variables (Fig. 4a and b), further analyses involving women with a BMI of 17 to 23 kg/m^2^ were performed (*n* = 1116; 78%). The GWG distribution was quite similar among the six pre-pregnancy BMI ranges (17 ≤ BMI < 18; 18 ≤ BMI < 19; 19 ≤ BMI < 20; 20 ≤ BMI < 21; 21 ≤ BMI < 22; and 22 ≤ BMI < 23) (Fig. 4c and d). However, the BW/GA percentile decreased with a decreasing pre-pregnancy BMI (Fig. 4e). A linear regression analysis showed the association between pre-pregnancy BMI and BW/GA percentile. The effect size of pre-pregnancy BMI was a 2.68% (95% CI, 1.55–3.81%) increase in BW/GA per 1 kg/m^2^ increase in pre-pregnancy BMI (*p* = 3.73 × 10^− 6^). To explore the cryptic heterogeneity of the GWG-BW/GA relationship, we hypothesized that the correlation between GWG and BW/GA differed according to newborn size. We then dichotomized the women into two groups according to the BW/GA percentile. Birth weight in Japan is generally low compared to world standards. Therefore, we also calculated the mean birthweight (SD) and the mean (SD) values of the international newborn size percentile for large and small sized newborns, according to INTERGROWTH-21st standards (larger-sized, 3258 (308), 52.0 (19.0); smaller-sized, 2719 (326), 13.4 (8.6)), suggesting that BW levels in the large half were equivalent to the world average levels but those in the small half were considerably low. A correlation between GWG and BW/GA in larger newborns was measured as r = 0.17 (*p* = 4.1 × 10^− 5^). In contrast, in smaller newborns, r = 0.099 (*p* = 1.9 × 10^− 2^). After adjustment for pre-pregnancy BMI, r = 0.18 (*p* = 1.2 × 10^− 5^) in larger newborns, whereas r = 0.092 (*p* = 2.9 × 10^− 2^) in smaller newborns. The disparity of the GWG and BW/GA correlation between large and small newborns indicated potential heterogeneity between them. Notably, there was a weaker correlation between GWG and BW/GA in women who delivered smaller newborns. We then further stratified women according to pre-pregnancy BMI (17 ≤ BMI < 18.5; 18.5 ≤ BMI < 20; 20 ≤ BMI < 23) (Additional file 5: Figure S2). The results showed that the GWG-BW/GA correlation disparity between the dichotomized groups existed across all BMI categories, including underweight women.

## Discussion

In this study, using sibling comparison, we found that an increase in GWG does not always contribute to an increase in BW/GA. Rather, BW/GA may be largely preset by familial factors before the perinatal period because the sibling correlation for BW/GA was found to be high. In addition, we found that the correlation between GWG and BW/GA was low in the women who delivered smaller children.

The overweight/obesity and excessive GWG significantly increase the risk of high birth weight, whereas underweight and inadequate GWG increase the incidence of low birth weight [9–18]. The prevalence of obesity in reproductive-aged women has increased worldwide in concordance with the increased prevalence in the general population, which varies among different countries [19]. Among different countries in Europe, North America, and Oceania, GWG was similar but differed according to BMI categories [20]. However, the distribution pattern of GWG stratified by BMI category largely varied among different ethnic groups [21]. It was shown that US-Japanese women had the lowest GWG compared to other ethnic groups residing in the USA, especially among underweight women [21]. Focusing on the situation in Japan, a large-scale nation-wide survey corroborated the high proportion of underweight (21.3%) and the low levels of GWG especially for underweight women [2]. From 1979 to 2010, a decrease in the mean birth weight occurred in parallel with an increase of underweight reproductive-aged women in Japan [22]. These data emphasize that major present problems in Japan are the high proportion of low-weight births and the high prevalence of underweight women, which is a striking contrast to what is occurring worldwide. Here, as a potentially modifiable factor, GWG and factors influencing GWG are important. Nutritional status including energy intake and dietary patterns may affect GWG levels as well as preconception weight. It is of note that the mean total calorie intake during pregnancy was far below the nationally recommended levels in Japan [23].

All the above studies basically used the linear-regression method to explore the effects of different maternal factors on birth weight. However, Koenker and Hallock [24] and Abrevaya [25] utilized a different approach, quantile regression, to analyze the effects of maternal demographics on birth weight in the USA. Whereas the linear regression estimates only pertain to how variables shift the mean of the birth weight distribution, the quantile regression method can provide information about how various factors impact different quantiles of the birth weight distribution. As such, this approach was utilized to quantify the effects of maternal race, education, weight gain, and prenatal care, etc., particularly for small fetuses (the lower fraction of birth weight distribution), attempting to reduce the rate of low-weight births [24, 25]. In addition, very recently, the quantile regression approach was appreciated and incorporated into the WHO study of fetal growth [26, 27], showing the graded effects of maternal factors on fetal growth. These ideas are consistent with our hypothesis that the effect of GWG on newborns varies depending on their size. Given these facts, it is important to determine how GWG impacts birth weight, especially in the lower fraction of birth weight in Japan. We also utilized quantile regression analysis to show that GWG had a weaker effect on birth weight for the smaller newborns (Additional file 6: Figure S3). This result verified the main result of the stratified analysis of the correlation between GWG and BW/GA. Of note, the effect of GWG was weaker for the lower fraction of the birth weight in Japan, although the previous study in the USA showed that the effect of GWG was stronger for the lower fraction [25].

Our findings have indicated the importance of considering neonatal size when attempting to determine the optimal GWG to reduce low birth weight. For example, if the child was healthy and large during the first pregnancy, and GWG during the second pregnancy is lower than expected, it might be possible to encourage an increase in weight gain within the recommended range. However, if the first child was healthy and small, careful consideration of whether to reinforce weight gain during the second pregnancy is necessary because GWG increase may not contribute to an increase in BW/GA. It would be safe to perform GWG management in consideration of the individual circumstances such as whether the women would probably deliver a large or small child, rather than uniformly recommending GWG increase. We have presented basic data for the above idea.

Our study has some strengths. The study population was well characterized and calculation of GWG was performed using the standard methodology. In addition, to the best of our knowledge, this is the first study to explore heterogeneity in the population in terms of a correlation between GWG and BW/GA. The prevention of low birth weight by uniform GWG increase is not yet an established method. The results of our study will be useful for devising more appropriate GWG recommendations.

Our study had several limitations. First, we dichotomized women according to BW/GA percentile to demonstrate heterogeneity: that some fetuses will respond to maternal weight gain and accordingly increase in weight while others will not respond. We hypothesized that heterogeneity exists between women who deliver large and small newborns; however, the true nature of heterogeneity is unknown. Since genetic polymorphisms [28, 29] and family environments [5, 28, 30] influence birth weight, such factors might be involved in generating heterogeneity. However, these factors are currently unidentified for Japanese pregnant women; therefore, we tentatively dichotomized according to BW/GA. We could at least accomplish our purpose to show the possibility of a difference in the GWG-BW/GA correlation between dichotomized groups. Future identification of the main factors for generating heterogeneity will improve the stratification method. Our study was performed as a single-center analysis; therefore, future studies involving a multi-center analysis are necessary to validate our results. Re-examining the effects of GWG on birth weight on a large scale by selecting low-birth-weight newborn populations may be necessary when defining the recommended GWG limit. In addition, the pre-pregnancy weights of the women were self-reported, which may have resulted in incorrect classifications of the GWG or pre-pregnancy BMI status. However, pre-pregnancy weights were mostly similar to the measured weights during early pregnancy and could be a satisfactory substitute for the recorded clinical data.

## Conclusions

Our study provides evidence that the correlation between GWG and BW/GA is dependent on neonatal size in Japan. Particularly, for low-birth-weight babies, it is notable that GWG increments do not always contribute to birth weight increases. However, we reproduced the positive association between GWG and birth weight for newborns whose weights were similar to the average neonatal birth weights based on world standards.

## Supplementary information

**Additional file 1: Table S1.** Distribution of child sex between the first and second deliveries

**Additional file 2: Table S2.** Distribution of small for gestational age, appropriate for gestational age, and large for gestational age between the first and second deliveries

**Additional file 3: Table S3.** Characteristics of the study population with unique individuals

**Additional file 4: Figure S1**. Inter-pregnancy difference in the BW/GA percentile

**Additional file 5: Figure S2.** Relationship between GWG and BW/GA percentile in the subgroups by pre-pregnancy BMI and BW/GA

**Additional file 6: Figure S3.** Graded effect of GWG on birth weight shown by quantile regression analysis.

## Data Availability

The datasets used and/or analyzed during the current study are available from the corresponding author on reasonable request.

## Abbreviations

BMI: Body mass index
BW/GA: Birth weight for gestational age
GWG: Gestational weight gain.
SGA: Small for gestational age
