# Supplementary material for: Stratified analysis of the correlation between gestational weight gain and birth weight for gestational age: a retrospective single-center cohort study in Japan
Source: BMC Pregnancy Childbirth. 2019 Nov 4;19:402. doi: 10.1186/s12884-019-2563-5 (PMC6829920; doi:10.1186/s12884-019-2563-5)
Supplement: Supplementary file 1 — Additional file 1: Table S1. Distribution of child sex between the first and second deliveries [file 12884_2019_2563_MOESM1_ESM.docx]

**Additional file 1 Table S1. Distribution of child sex between first and second deliveries**

|  | | Second delivery | |
| --- | --- | --- | --- |
|  |  | Female | Male |
| First delivery | Female | 15 (20) | 19 (25) |
|  | Male | 24 (32) | 17 (23) |

Data are N (%).
