# Supplementary material for: Stratified analysis of the correlation between gestational weight gain and birth weight for gestational age: a retrospective single-center cohort study in Japan
Source: BMC Pregnancy Childbirth. 2019 Nov 4;19:402. doi: 10.1186/s12884-019-2563-5 (PMC6829920; doi:10.1186/s12884-019-2563-5)
Supplement: Supplementary file 2 — Additional file 2: Table S2. Distribution of small for gestational age, appropriate for gestational age, and large for gestational age between the first and second deliveries [file 12884_2019_2563_MOESM2_ESM.docx]

**Additional file 2 Table S2. Distribution of small for gestational age, appropriate for gestational age, and large for gestational age between first and second deliveries**

|  | | Second delivery | | |
| --- | --- | --- | --- | --- |
|  |  | SGA | AGA | LGA |
| First delivery | SGA | 0 (0) | 5 (6.7) | 0 (0) |
|  | AGA | 5 (6.7) | 57 (76) | 2 (2.7) |
|  | LGA | 0 (0) | 6 (8.0 | 0 (0) |

Data are N (%). SGA, small for gestational age; AGA, appropriate for gestational age; LGA, large for gestational age.
