# Supplementary material for: Stratified analysis of the correlation between gestational weight gain and birth weight for gestational age: a retrospective single-center cohort study in Japan
Source: BMC Pregnancy Childbirth. 2019 Nov 4;19:402. doi: 10.1186/s12884-019-2563-5 (PMC6829920; doi:10.1186/s12884-019-2563-5)
Supplement: Supplementary file 3 — Additional file 3: Table S3. Characteristics of the study population with unique individuals [file 12884_2019_2563_MOESM3_ESM.docx]

**Additional file 3 Table S3. Characteristics of the study population with unique individuals**

|  | **Pre-pregnancy BMI < 17 (n =52)** | **17 ≤ Pre-pregnancy BMI < 23 (n = 1116)** | **23 ≤ Pre-pregnancy BMI (n = 272)** |
| --- | --- | --- | --- |
| Maternal age (year), mean (SD) | 31.8 (4.2) | 33 (4.5) | 34.1 (4.9) |
| Maternal height (cm), mean (SD) | 161.1 (5.4) | 159 (5.4) | 158.6 (5) |
| Maternal weight (kg), mean (SD) | 42.4 (3.4) | 50.4 (4.9) | 65.4 (9.3) |
| Pre-pregnancy BMI (kg/m^2^), mean (SD) | 16.3 (0.7) | 19.9 (1.5) | 26 (3.4) |
| Multiparity, N (%) | 17 (33) | 360 (32) | 85 (31) |
| Gestational week of delivery, mean (SD) | 39.2 (1.1) | 39.3 (1.5) | 39.4 (1.4) |
| Gestational weight gain (kg/40 weeks), mean (SD) | 11.1 (3.6) | 10.6 (3.2) | 8.7 (4.5) |
| Child sex (male), N (%) | 29 (56) | 29 (56) | 29 (56) |
| Birthweight (g), mean (SD) | 2942 (353) | 2989 (416) | 3115 (411) |
| Birth length (cm), mean (SD) | 49.4 (1.6) | 49.5 (2.2) | 49.9 (1.9) |
| Head circumference (cm), mean (SD) | 33.2 (1.1) | 33.4 (1.4) | 33.8 (1.3) |
| Chest circumference (cm), mean (SD) | 31.8 (1.4) | 31.8 (1.8) | 32.4 (1.7) |
| BW/GA percentile, mean (SD) | 47.8 (27.7) | 50.6 (28.3)* | 61 (28.2) |
| SGA, N (%) | 4 ( 7.7) | 99 (8.9) | 14 (5.1) |

Data are mean (SD) or N (%). *n = 1114 because two deliveries were post-term.
