# Supplementary material for: Stratified analysis of the correlation between gestational weight gain and birth weight for gestational age: a retrospective single-center cohort study in Japan
Source: BMC Pregnancy Childbirth. 2019 Nov 4;19:402. doi: 10.1186/s12884-019-2563-5 (PMC6829920; doi:10.1186/s12884-019-2563-5)
Supplement: Supplementary file 4 — Additional file 4: Figure S1. Inter-pregnancy difference in the BW/GA percentile [file 12884_2019_2563_MOESM4_ESM.docx]

**Additional file 4 Figure S1. Inter-pregnancy differences in the BW/GA percentile**

1. Relationship between inter-pregnancy differences in the GWG and BW/GA percentile. X-axis denotes the differences between the second GWG and the first GWG. Y-axis denotes the differences between the second BW/GA percentile and the first BW/GA percentile.
2. Histogram of the absolute value of the inter-pregnancy differences in the BW/GW percentile.

GWG, gestational weight gain; BW/GA, birth weight for gestational age.
