# Supplementary material for: Stratified analysis of the correlation between gestational weight gain and birth weight for gestational age: a retrospective single-center cohort study in Japan
Source: BMC Pregnancy Childbirth. 2019 Nov 4;19:402. doi: 10.1186/s12884-019-2563-5 (PMC6829920; doi:10.1186/s12884-019-2563-5)
Supplement: Supplementary file 5 — Additional file 5: Figure S2. Relationship between GWG and BW/GA percentile in the subgroups by pre-pregnancy BMI and BW/GA [file 12884_2019_2563_MOESM5_ESM.docx]

**Additional file 5 Figure S2. Relationship between GWG and BW/GA percentile in the subgroups by pre-pregnancy BMI and BW/GA**


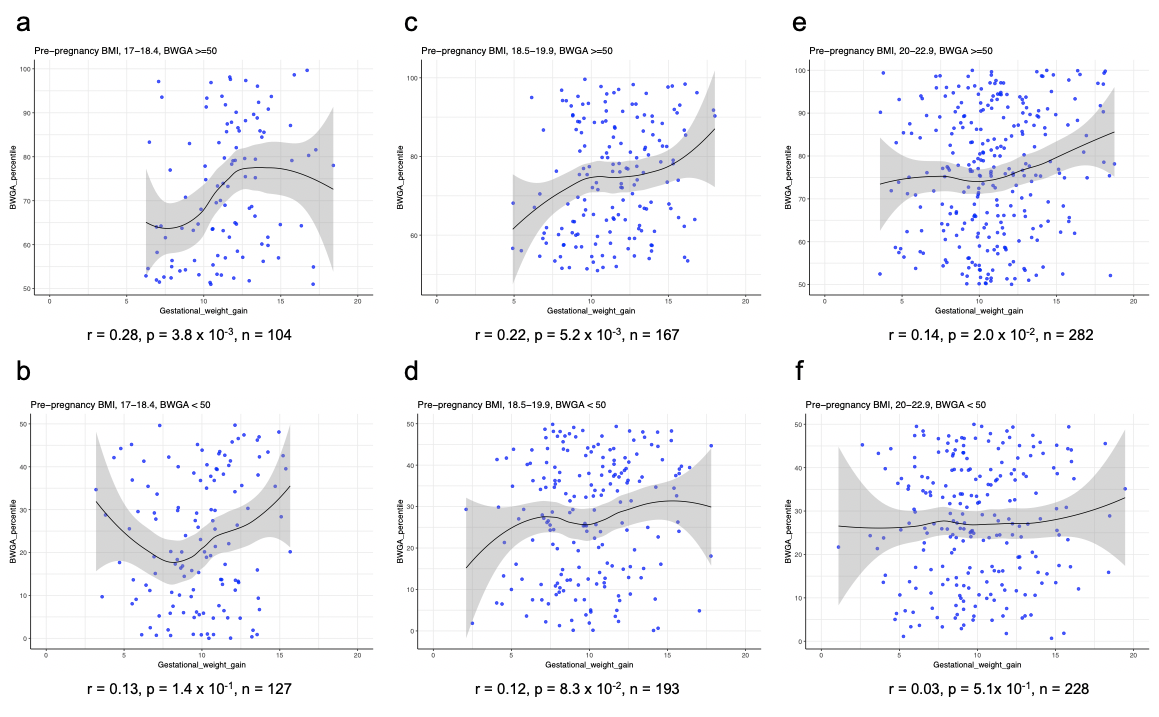


Scatter plots showing the relationship between GWG and the BW/GA percentile for distinct groups stratified by pre-pregnancy BMI and neonatal size. (a, b) 17 ≦ pre-pregnancy BMI < 18.5; (c,d) 18.5 ≦ pre-pregnancy BMI < 20; (e,f) 20 ≦ pre-pregnancy BMI < 23; (a,c,e) 50 ≦ BW/GA percentile; (b,d,f) BW/GA percentile < 50. Women with extremely high and low GWG (outside the first quartile -1.5 x interquartile range and the third quartile + 1.5 x interquartile range) were excluded. Correlation coefficient between GWG and BW/GA (r), p-value, and sample size (n) are indicated. Black line with gray band indicates the local polynomial regression line with standard error. BW/GA, birth weight for gestational age.
