# Supplementary material for: Stratified analysis of the correlation between gestational weight gain and birth weight for gestational age: a retrospective single-center cohort study in Japan
Source: BMC Pregnancy Childbirth. 2019 Nov 4;19:402. doi: 10.1186/s12884-019-2563-5 (PMC6829920; doi:10.1186/s12884-019-2563-5)
Supplement: Supplementary file 6 — Additional file 6: Figure S3. Graded effect of GWG on birth weight shown by quantile regression analysis. [file 12884_2019_2563_MOESM6_ESM.docx]

**Additional file 6 Figure S3. Graded effect of GWG on birth weight shown by quantile regression analysis**

The effect of GWG on birth weight percentiles (represented by quantiles; 0.1-0.9 quantiles correspond to 10-90 percentiles) is shown, which was estimated by quantile multivariate regression analysis, using the quantreg R package and applied to the same main cohort used for dichotomization in the text. In the model used, the predictor variables were gestational age, fetal sex, parity, pre-pregnancy BMI, and GWG and the outcome was birthweight. The grey zone indicates 95% CI. Linear regression estimates (solid) with 95% CI (dashed) are shown in red. GWG, gestational weight gain; BMI, body mass index; CI, confidence interval.
